# Differential regulation of spermatogenic process by *Lkb1* isoforms in mouse testis

**DOI:** 10.1038/cddis.2017.527

**Published:** 2017-10-12

**Authors:** Feifei Kong, Mei Wang, Xiaojing Huang, Qiuling Yue, Xiang Wei, Xiaowei Dou, Xiaoxu Peng, Yuanyuan Jia, Ke Zheng, Tinghe Wu, Jun Yan, Jing Li

**Affiliations:** 1 State Key Laboratory of Reproductive Medicine, Nanjing Medical University, Nanjing 210029, China; 2 State Key Laboratory of Pharmaceutical Biotechnology and MOE Key Laboratory of Model Animals for Disease Study, Model Animal Research Center, Nanjing University, Nanjing 210008, China; 3 Department of Biotechnology and Biomedicine, Yangtze Delta Region Institutes of Tsinghua University, Jiaxing 314006, China; 4 Collaborative Innovation Center for Genetics and Development, Shanghai 200438, China

## Abstract

Liver serine/threonine kinase B1 (LKB1) is a tumor suppressor associated with the pathogenesis of Peutz-Jeghers syndrome. Affected males are at increased risk of developing Sertoli cell tumors and display defective spermatogenesis. Male mice lacking the short isoform (*Lkb1*_*S*_) of *Lkb1* were sterile and exhibited abnormal spermiogenesis. In addition to the short isoform, the long isoform of *Lkb1* (*Lkb1*_*L*_) is also expressed in testis; however, the requirement of the long isoform for fertility and the functional difference between the isoforms remain unknown. Herein, different from the spermiation failure reported in *Lkb1*_*S*_ knockout mice, conditional deletion (cKO) of both isoforms of *Lkb1* in germ cells resulted in male sterility stemming from defects in acrosome formation, as well as nuclear elongation and condensation during spermatid differentiation. Additionally, cKO mice showed a progressive germ cell loss that was never reported in mice with *Lkb1*_*S*_ deletion. Further experiments revealed that the defect resulted from the failure of spermatogonial stem/progenitor cells (SPCs) maintenance. Although increased mTORC1 activity in postnatal cKO testes was consistent with a tendency toward germline stem cell differentiation, *in vivo* inhibition of the pathway by rapamycin treatment failed to rescue the phenotype. Concurrently, we detected a significant reduction of mitochondrial activity in *Lkb1*deficient SPCs. The results suggest that the regulation of LKB1 on SPCs’ maintenance is associated with mitochondrial functions but not through the mTOR signaling pathway. In summary, our study supports different roles of *Lkb1* isoforms in spermatogenesis with *Lkb1*_*L*_ directing SPCs maintenance, and *Lkb1*_*L*_ and *Lkb1*_*S*_ coordinately regulating spermatid differentiation.

Liver kinase B1 (*Lkb1*), also known as Serine/Threonine protein kinase 11 (*Stk11)*, encodes an evolutionarily conserved serine/threonine kinase required in many physiological processes.^1^ The well-known downstream substrates of LKB1 are AMP-activated protein kinase (AMPK), and 12 other AMPK-related kinases. Tremendous progress over the last few years revealed that LKB1 negatively regulates mammalian target of rapamycin complex 1 (mTORC1) activity through its substrate AMPK, and the loss of LKB1 leads to the aberrant activation of mTORC1 in a variety of tissues.^2^ In brief, the LKB1/AMPK/mTORC1 cassette constitutes a canonical signaling pathway that integrates information on the metabolic and nutrient status to regulate many biological processes including cell proliferation, cell survival and autophagy.

Many studies have shown the tissue-specific functions of *Lkb1* in pancreas, skin, muscle and gastrointestinal tissues.^3, 4, 5^ In testis, the function of LKB1 was not clearly described until knockout of an alternate splice variant was reported to cause sterility in mice.^6^ The newly identified isoform was termed LKB1 short form (LKB1_S_) as opposed to the previously reported long form (LKB1_L_).^6^ The two proteins are identical except for the C-terminus, which is encoded by different exons, 9A *versus* 9B for LKB1_L_ and LKB1_S_, respectively and thus differ in length. Although both isoforms are widely expressed in rodent and human tissues, *Lkb1*_*S*_ is particularly abundant in haploid spermatids.^7^ Male mice that cannot generate *Lkb1*_*S*_are sterile with abnormal release of mature spermatids from the seminiferous epithelium.^7, 8^ Spermatogenesis is a unique physiological process, during which diploid spermatogonial stem/progenitor cells (SPCs) undergo a series of differentiated steps, followed by meiosis and spermatid differentiation, and culminate in the production of mature haploid sperm cells. Although *Lkb1*_*S*_ is the dominant isoform in germ cells (round spermatids),^8^ it remains unknown if the *Lkb1*_*L*_ isoform is expressed in germ cells and if the two isoforms function redundantly or indispensably during the process of spermatogenesis.

Herein, we report the differential expression of the two isoforms of *Lkb1* in different spermatogenic cell types with *Lkb1*_*S*_ mainly expressed in spermatids and *Lkb1*_*L*_ in SPCs, respectively. After conditional deletion (cKO) of *Lkb1* in male germ cells, mice were sterile as evidenced by severe defects on round spermatid differentiation and flagellum biogenesis. Meanwhile, these cKO mice also suffered a progressive loss of germ cells, resulting in a Sertoli-cell-only phenotype. Further experiments demonstrated the essential roles of Lkb1 in SPCs maintenance and spermatid differentiation.

## Results

### Distinct expression of *Lkb1*_*L*_and *Lkb1*_*S*_ in different spermatogenic cell types

**Figure 1:**
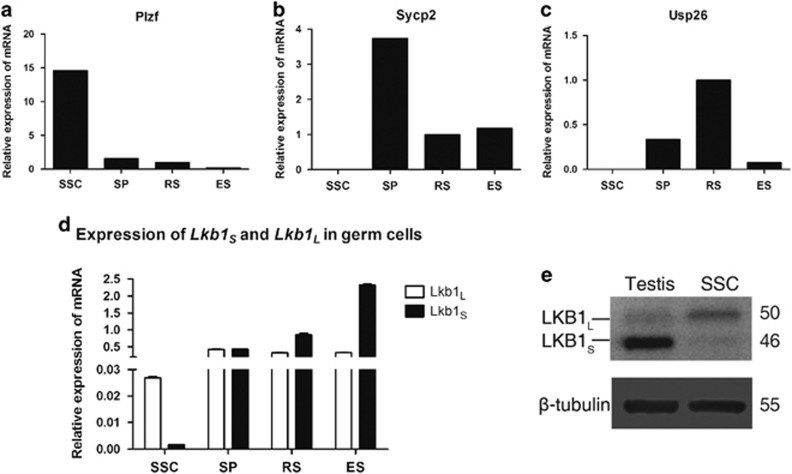
Expression of *Lkb1*_*L*_ and *Lkb1*_*S*_ in different spermatogenic cell types. Spermatogenic cells at different developmental stages (SP – spermatocytes, RS – round spermatids and ES – elongated spermatids) were sorted from pooled adult testes via STA-PUT velocity sedimentation. Mouse SSCs were enriched from P8 testes and cultured *in vitro* for at least five passages. All experiments were performed with at least three replicates. (**a**–**c**) The purity of these cells was confirmed by RT-PCR using markers: (**a**) *Plzf* for SSC, (**b**) *Sycp2* for SP, (**c**) *Ups26* for CS and ES. (**d**) RT-PCR for *Lkb1*_*L*_ and *Lkb1*_*S*_transcripts in each spermatogenic cell type. (**e**) Western blot analysis of Lkb1_*L*_ and Lkb1_*S*_in adult testis and SSCs

To delineate the roles of LKB1 in male germ cells during spermatogenesis, we first examined the expression of the two isoforms of *Lkb1* in the different germ cell stages including spermatogonial stem cells (SSC), spermatocytes (SP), round spermatids (RS) and elongated spermatids (ES). The purity of each isolated spermatogenic cell type was confirmed by enrichment assay on their corresponding marker gene expression, such as *Plzf* for SSCs, *Sycp2* for SPC and *Usp26* for RS and ES (Figure 1a–c).^9, 10, 11^ *Lkb1*_*S*_ was predominantly expressed in differentiated germ cells (SP, RS and ES) and a marked increase was observed in RS and ES. *Lkb1*_*L*_ was expressed in these cell types but maintained a stable expression level. Unlike *Lkb1*_*S*_, *Lkb1*_*L*_ was detected at a considerable level in SSCs, albeit its expression level was lower than that in other cell types (Figure 1d). Western blot analysis further demonstrated that LKB1_S_ is the main isoform expressed in testis, while LKB1_L_ is the main isoform in SSCs (Figure 1e). The developmentally differential expression pattern of two *Lkb1* isoforms points to their potentially discrepant regulatory functions in spermatogenesis.

### Defects of spermiogenesis in *Lkb1* cKO mice

**Figure 2:**
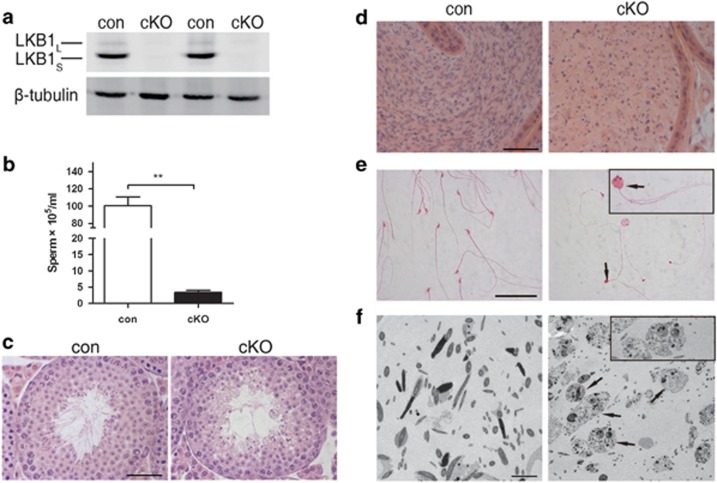
Effect of conditional deletion (cKO) of *Lkb1* in germ cells on spermatogenesis and epididymal sperm morphology. Testes were collected from control (con) and cKO mice for western blot and H&E staining at 5 weeks of age. Sperm were collected from cauda epididymides at 13 weeks of age for analysis. All experiments were replicated at least three times. (**a**) Significant decrease of Lkb1 proteins in *Lkb1* cKO mice. (**b**) Sperm count from cauda epididymides. **P<0.01. (**c**) Representative tubules in 5-week-old control and cKO testes. Scale bar=50 *μ*m. (**d**) Histology of epididymal tubules and sperm analysis. Scale bar=50 *μ*m. (**e**) Spermatozoa morphology in control and cKO mice. Black arrows, abnormal multinucleated and multiflagellated sperm. Scale bar=50 *μ*m. (**f**) Transmission electron microscopy of sperm in the epididymis. Black arrows and inset, multinucleated sperm. Scale bar=5 *μ*m

To study the functions of *Lkb1* in germ cells, we conditionally deleted *Lkb1* in the male germ line by means of *Vasa-Cre* (*Ddx4-Cre*) and the flox’d allele *Lkb1*^*loxp*^.^12^ The result of western blot confirmed the mutation in cKO testis (Figure 2a). As expected, *Lkb1* cKO male mice were infertile. The average sperm count in cKO mice was 3.4 × 10^5^ spermatozoa per epidydimis, which accounted for a >96% reduction as compared with control group (Figure 2b). Moreover, the sperm heads were invariably round with most displaying multi-nucleation and/or multi-flagella (Figure 2e). Upon histological evaluation, in cKO mice the epididymal lumen was filled with sloughed germ cells or cell debris (Figure 2d). Transmission electron microscopy further demonstrated that this debris consisted of degraded multinucleated germ cells (Figure 2f). Testis sections from 5-week old control and cKO mice were used to analyze the first wave of spermatogenesis. In control mice, mature spermatozoa observed at the luminal interface. By contrast, multinucleated giant round cells were frequently observed towards the center of the tubule in cKO mice (Figure 2c). These results suggest that the dramatic reduction of spermatozoa in the epidydimis stemmed from a failure in the process of spermiogenesis.

### Lack of acrosome formation in *Lkb1* cKO mice

**Figure 3:**
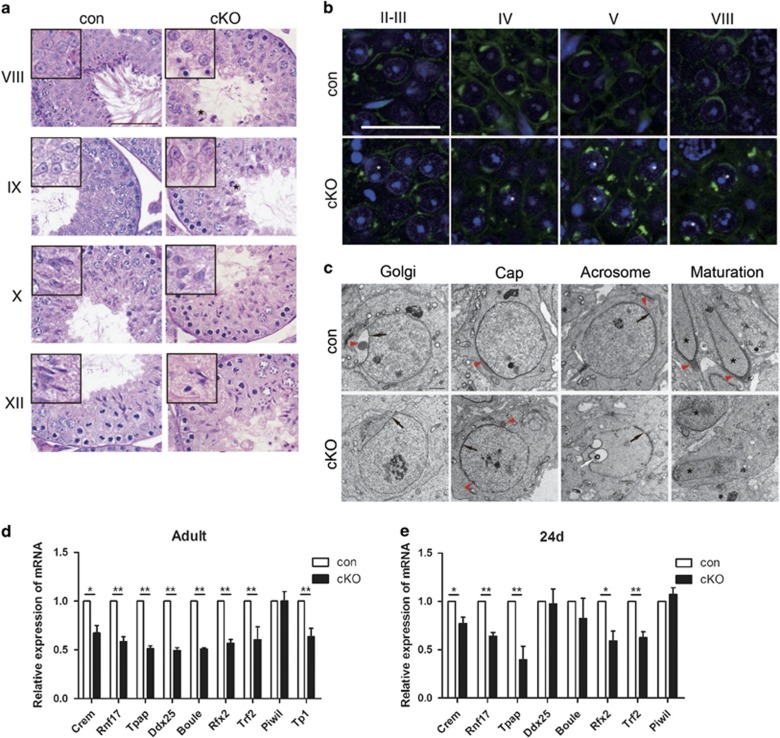
Abnormal acrosomal biogenesis in *Lkb1* cKO mice. Testes from both control and cKO mice (*n*=3) were collected at 6 weeks of age for tubule analysis. (**a**) Representative images of control and comparable cKO testis sections are shown at developmental stages I–VIII, IX, X and XII. Asterisks mark giant and round cells. Scale bars=50 *μ*m. (**b**) Higher magnification of acrosomes stained by fluorescently tagged peanut agglutinin at different stages of spermatogenesis. Scale bars=25 *μ*m. Asterisks, round spermatid with multiple pre-acrosomal vesicles. (**c**) Ultrastructural analysis of acrosome formation in control and *Lkb1* cKO spermatids. Arrowheads, acrosomal granules or acrosomes. Black arrows, closer leaflets of nuclear envelope. White arrows, developing axonemes or centriole-like organelle. Black asterisks, spermatid nuclei. Scale bar=1 *μ*m. (**d** and **e**) RT–PCR analysis for spermiogenesis-related key regulator genes in adult (**d**) and P24 (**e**) control and cKO mice. Data are presented as means ± S.E.M. of at least three replicates. *P<0.05 and **P<0.01, compared with control mice. The level of mRNA expression in control mice was given an arbitrary value of 1

In mammals, spermiogenesis is characterized by a massive wave of transcriptional activity and can be accurately divided into continuous steps on the basis of nuclear elongation and acrosome morphology.^13^ To further assess potential defects in spermiogenesis stemming from the *Lkb1* cKO phenotype, we first compared spermatid nuclear elongation between similarly staged seminiferous tubules from control and cKO mice at 6 weeks of age. At stages from I to VIII, round spermatids with a spherical, central nucleus were commonly observed in both control and cKO mice. However, in stages IX and X, progressive elongation and condensation of spermatid nucleidid did not occur in cKO tubules (Figure 3a). We next used fluorescently tagged peanut agglutinin to label and follow acrosome development. In rodents, acrosome development begins early in the spermiogenic process and can be divided into four phases (Golgi, Cap, Acrosome and Maturation) comprising the transformation from round spermatids to mature spermatozoa.^14^ The Golgi and Cap phases, which encompass the formation of a large acrosome granule (Golgi) and its growth and flattening over the nucleus (Cap phase), were observed in stages II–VIII round spermatids from control mice (Figure 3b, upper panel). However, only occasionally, two pre-acrosomal vesicles could be observed associated with the nucleus in stages II–III round spermatids from cKO mice (Figure 3b, lower panel). Moreover, Cap flattening was seldom observed in stages IV–VIII cKO tubules and, instead, pre-acrosomal granules failed to fuse and appeared scattered over the spermatid nucleus (Figure 3b, lower panel). TEM analysis revealed the typical acrosomal granule and cap structure over round spermatids at Golgi phases in control mice (Figure 3c). Then, cap flattening continued through acrosome-acroplaxome complex formation and culminating in acrosome development. Although the closer leaflets of nuclear envelope could be identified at the beginning of the acrosome formation in cKO mice (Figure 3c, black arrows), it lacked a typical large acrosomal granule; furthermore, it failed to form the cap structure and instead displayed small granules scattered and attached over the nuclear membrane in Cap phase (Figure 3c, red arrowheads, cKO *versus* control). Additionally, localization of the centriole distally to begin formation of the flagellum as observed by Acrosome phase in control tubules was not observed in spermatids from cKO spermatids (Figure 3c, white arrow). Taken together, the failure of acrosome and flagellum genesis observed in round spermatids impaired nuclear elongation and remodeling and ultimately lead to abnormal spermatozoal maturation in cKO mice.

At the round spermatid stage, a large number of testis-specific genes are expressed under the control of a limited number of key regulator genes, including *Crem*, *Trf2*, *Rnf17*, *Piwil1*, *Boule*, *Tpap*, *Ddx25* and *Rfx2*.^15, 16, 17, 18, 19, 20, 21, 22, 23, 24^ To investigate the potential role of *Lkb1* in spermiogenesis regulation, we compared the mRNA expression of these key regulator genes in adult testes of control and cKO mice. Notably, in cKO testes there was a global significant decrease in the expression of all genes examined except *Piwil1* (Figure 3d). Then, to rule out whether the decreased gene expression levels stemmed directly from faulty transcriptional regulation by Lkb1 rather than the altered ratio of cell types in adult testis, we repeated these experiments in testes from 24-day-old mice, tubules from both control and cKO mice presented a similar histomorphology (Supplementary Figures S1a,b). In agreement, at this age, cKO mice also showed a significant downregulation of most genes examined (Figure 3e). Altogether, these results suggest that loss of Lkb1 may disrupt spermatid gene transcription.

### Progressive loss of germ cells in cKO mice

**Figure 4:**
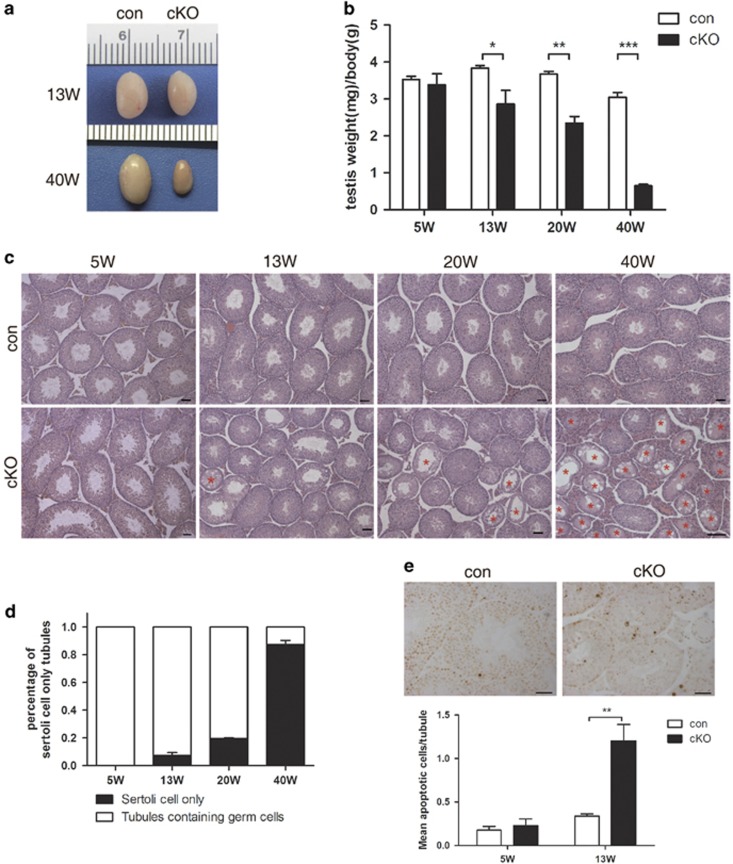
Gradual germ cell loss in *Lkb1* cKO mice. Testes were collected from control and cKO mice (*n*=3–5) at 5, 13, 20 and 40 weeks of age. (**a**) Testis gross appearance at 13 and 40 weeks of ages in both control and cKO mice. (**b**) Testis weight per body weight. (**c**) Histology of the seminiferous tubules. Scale bars=50 *μ*m. Red asterisks, Sertoli only tubules. (**d**) Percentage of Sertoli only tubules in each section. (**e**) Upper panel, TUNEL staining of seminiferous tubules in 13W control and cKO mice. Lower panel, average number of apoptotic cells per tubule in 5- and 13-week-old testes by TUNEL staining. All data presented as mean±S.E.M. *P<0.05; **P<0.01

While a link between *Lkb1*_*S*_KO and male sterility resulting from spermiogenetic defects was previously reported, no phenotypes were reported on the other stages of spermatogenesis.^8^ In this study, we recorded a gradual decrease in testis weight with advancing age (Figures 4a and b). Histology analysis also showed a progressive loss of germ cells in seminiferous tubules that finally resulted in Sertoli-only tubules (Figure 4c). The loss of spermatogenic cells began after 5 weeks of age and, by 13 weeks, tubules undergoing various degrees of germ cell loss were widespread in cKO testis’ sections (Figure 4c; Supplementary Figures S1c–g). By 40 weeks, cKO testes became completely atrophic and nearly 90% were Sertoli-only tubules (Figure 4d). These results are consistent with a normal onset of spermatogenesis in juvenile cKO mice followed by an age-dependent progressive deletion of spermatogenic cells from the seminiferous tubules.

### Depletion of SPCs in *Lkb1* cKO mice

**Figure 5:**
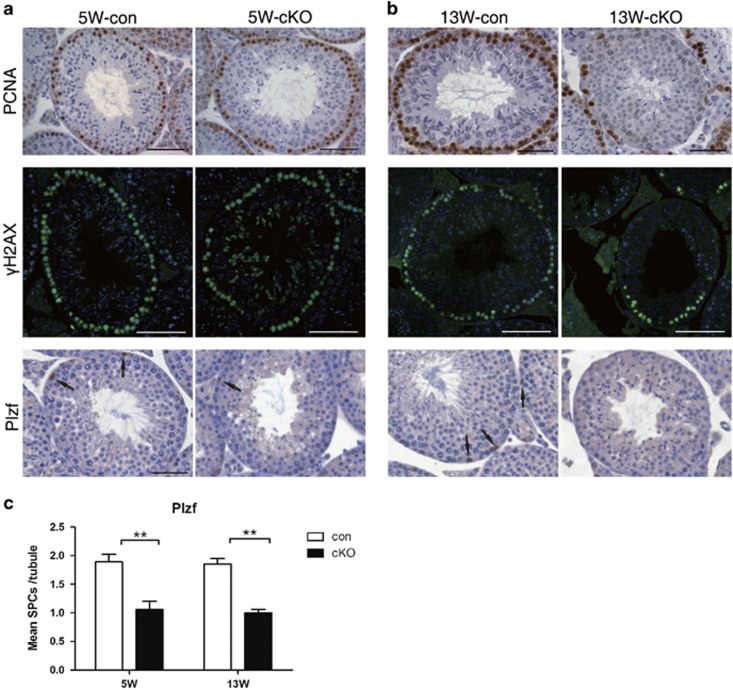
Depletion of SPCs in *Lkb1* cKO mice. Seminiferous tubules from 5- (**a**) and 13- (**b**) week-old control and *Lkb1* cKO mice (*n*=3) were used for immunostaining using specific antibodies against PCNA, *γ*H2AX and Plzf. Scale bars=50 *μ*m. Arrows, representative Plzf-positive cells in tubules. (**c**) Quantification of Plzf-positive germ cells in each tubule. Data are presented as mean±S.E.M. **P < 0. 01

To investigate the underlying cause for the scarcity of spermatogenic cells observed in cKO seminiferous tubules, we used the TUNEL assay to assess apoptosis in 5- and 13-week-old control and cKO testes. Although apoptotic cell counts increased in 13-week cKO testis, numbers were still low (~1.2 apoptotic cells per tubule) and there was no preference for any specific cell type (Figure 4e). These results suggested that the testicular atrophy observed in cKO mice was not caused by induction of apoptosis. We then used stage-specific spermatogenic cells markers to assess at which developmental stage germ cell loss was happening. These markers included: PCNA, a proliferation marker confined to the nuclei of spermatogonia and early spermatocytes;^25^ *γ*H2AX, in spermatocytes in meiotic prophase I;^26^ and Plzf, a SPC marker.^27^ In control testis, PCNA (Figures 5a and b, upper panel) and *γ*H2AX (Figures 5a and b, middle panel) were evenly and continuously distributed around the seminiferous tubules. Intriguingly, PCNA and *γ*H2AX staining was comparable to that of controls in 5-week-old cKO mice (Figure 5a), but both of them decreased significantly by 13 weeks in cKO testes (Figure 5b). Strikingly, in opposition to the observations with PCNA and *γ*H2AX labeling, the number of Plzf-positive cells was reduced as early as 5 weeks in cKO mice (Figures 5a and b). These results suggest that progressive depletion of SPCs is at the origin of germ cell loss in cKO mice.

### The indispensable role of *Lkb1* in SPC self-renewal and maintenance

**Figure 6:**
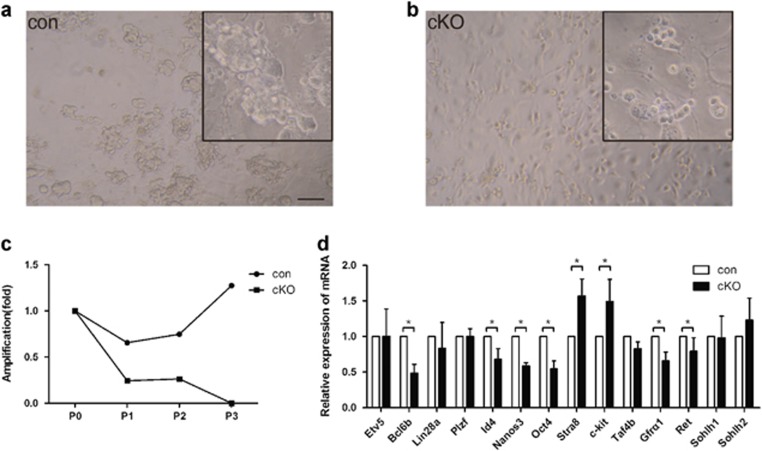
Requirement for *Lkb1* in SPC maintenance. Testes from P8 control and cKO mice (*n*=2–4) were used for SPCs enrichment and culture. (**a**,**b**) Microscopic morphology of presumptive SSCs derived from P8 control **(a**) and *Lkb1* cKO (**b**) mice, following *in vitro* culture at passage 2. Insets, enlarged view of cells in culture. The culture was repeated for two times. Scale bar=200 *μ*m. (**c**) Amplification curve of control and *Lkb1* cKO SPCs cultured *in vitro*. *y* axis represents relative increase in the number of SPCs after subcloning (fold change of initially plated cell numbers in culture). (**d**) Comparative expression of spermatogonial self-renewal and differentiation-related genes in freshly isolated SPCs from control and cKO littermates (*n*=3). The data were presented as mean±S.E.M. of at least three replicates. The level of mRNA expression in control mice was given an arbitrary value of 1. *P<0.05; **P<0.01

A major advance in the study of male germline biology was the development of a culture system allowing long-term maintenance and expansion of mouse SSCs by *in vitro* culture.^28^ To investigate the effects of *Lkb1* deletion on SSC survival and maintenance, we enriched SPCs using Thy-1 microbeads according to previous reports.^29^ Similar numbers of cells were separated and seeded onto STO feeder cells for culture. To our surprise, the *Lkb1* cKO germ cells failed to form typical SSC colonies in culture (Figure 6b). After two passages, the *Lkb1* cKO germ cells underwent complete depletion (Figure 6c). Freshly enriched SPCs from control and cKO testis were then used to evaluate the expression of SSC self-renewal and differentiation-related genes by RT-PCR. As shown in Figure 6d, key regulators of SSC self-renewal including Oct4, *Bcl6b, Nanos3, Id4,* and *Gfrα1* displayed decreased expression in *Lkb1* cKO germ cells.^30, 31, 32^ Conversely, the expression of the SSC differentiation markers, *Stra8* and *c-kit* was significantly upregulated in this population.^33, 34^

### The depletion of SPCs in cKO mice is independent of the mTOR signaling pathway but associated with mitochondria dysfunction

**Figure 7:**
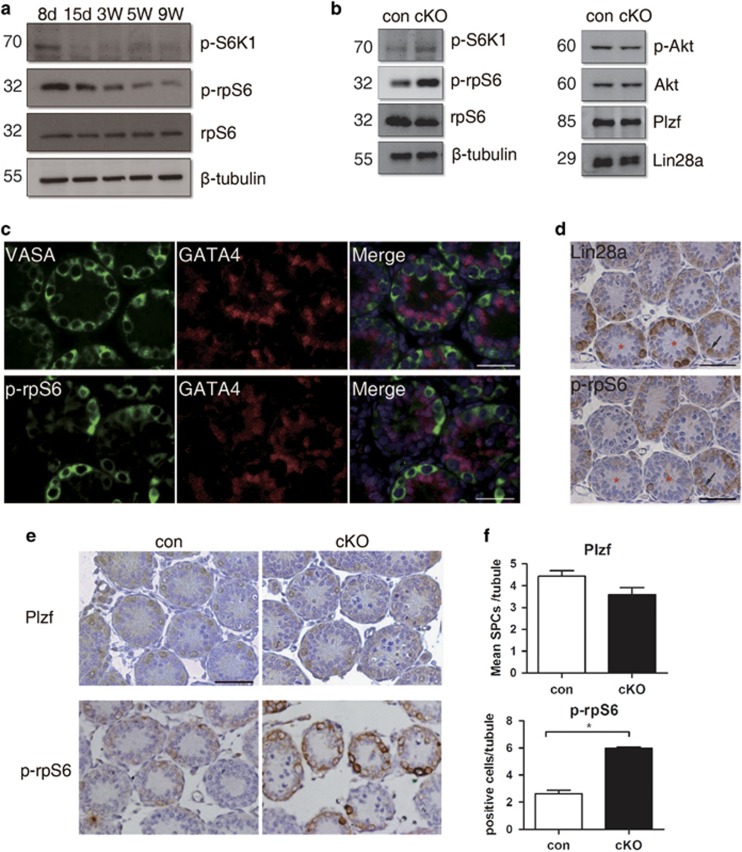
Increased mTORC1 activity in germ cells from *Lkb1* newborn cKO testis. (**a**) Western blot of testis proteins at different postnatal ages using specific antibodies against p-S6K1 (T389), p-rpS6 (S235/6), rpS6 and *β*-tubulin. rpS6, and *β*-tubulin were used as internal controls. (**b**) Expression of PI3K-, mTOR- and SPC-related markers by western blot in P8 testes from control and *Lkb1* cKO mice. (**c**) Immunohistochemistry for VASA, GATA4, p-rpS6 in P8 control testis. (**d**) Immunohistochemistry for Lin28a and p-rpS6 in P8 control testis. Serial sections were used in order to compare cellular localizations of these markers in the same tubule. Asterisks, different distribution of Lin28a and p-rpS6 in the same tubule resulting in strong positive for Lin28a and weak staining for p-rpS6. Arrows, cells expressing both Lin28a and p-rpS6. (**e**) Immunohistochemistry for Plzf and p-rpS6 in control and cKO mice. (**f**) Mean number of Plzf- or p-rpS6-positive cells in each tubule (positive cells/total tubules). The data were shown as mean±S.E.M. of at least three replicates. *P<0.05. Scale bars=50 *μ*m

It has been reported in a variety of tissues that the inhibition of LKB1 signaling induced the aberrant activation of the mTOR signaling pathway.^35, 36, 37^ As a central signaling pathway in stem cell homeostasis, the aberrant activation of mTORC1 was recently shown to promote SSC differentiation in both mouse and *Drosophila*.^35, 38, 39^ Since RT-PCR revealed an increased expression of differentiation markers *Stra8* and *c-kit* in freshly isolated SSCs from cKO mice, we assessed whether the mTOR signaling pathway was functioning downstream of LKB1 in regulating SSCs maintenance and differentiation in our model. Testes were collected at different age and the highest mTORC1 activity was detected at P8 (Figure 7a). We then used P8 testes to compare mTORC1 signaling between control and cKO mice. As shown in Figure 7b, *Lkb1*-deficient germ cells induced a significant increase in phosphorylated S6K1 and rpS6, but no effect on p-Akt, a marker of the PI3K signaling pathway. Additionally, Lin28 and Plzf showed similar expression in both control and cKO P8 testes at mRNA and protein levels (Figures 6d and 7b). To investigate the origin of the aberrant activation of the mTOR signaling pathway, we then checked the localization of p-rpS6 on sections of control P8 testis using VASA as a germ cell marker, GATA4 as a Sertoli cell marker and Lin28a, as a SPC marker. From the localization of VASA, p-rpS6 and GATA4, it was confirmed that the activation of mTORC1 was restricted to germ cells and not all VASA-positive germ cells are p-rpS6 positive (Figure 7c). Then we used Lin28a to label SPCs in the tubules and found p-rpS6 and Lin28a seldom co-localized in the same germ cell when serial sections were used for staining. Moreover, even when they were expressed in the same germ cell, the signal for both p-rpS6 and Lin28a was always lower than that of when expressed separately (Figure 7d, arrows). Because Lin28a expressed in both undifferentiated and differentiating spermatogonia,^40^ we then used Plzf to label undifferentiated germ cells and Plzf- and p-rpS6-positive cells were counted (Figure 7e). While there was only a little decrease of Plzf-positive germ cells in P8 cKO testes, p-rpS6-positive cells increased significantly in cKO testes with nearly twice as many as those in controls (Figure 7f). These results suggest that the abnormal higher activity of the mTOR signaling pathway may be related to the acceleration of spermatogonial differentiation in cKO testis.

**Figure 8:**
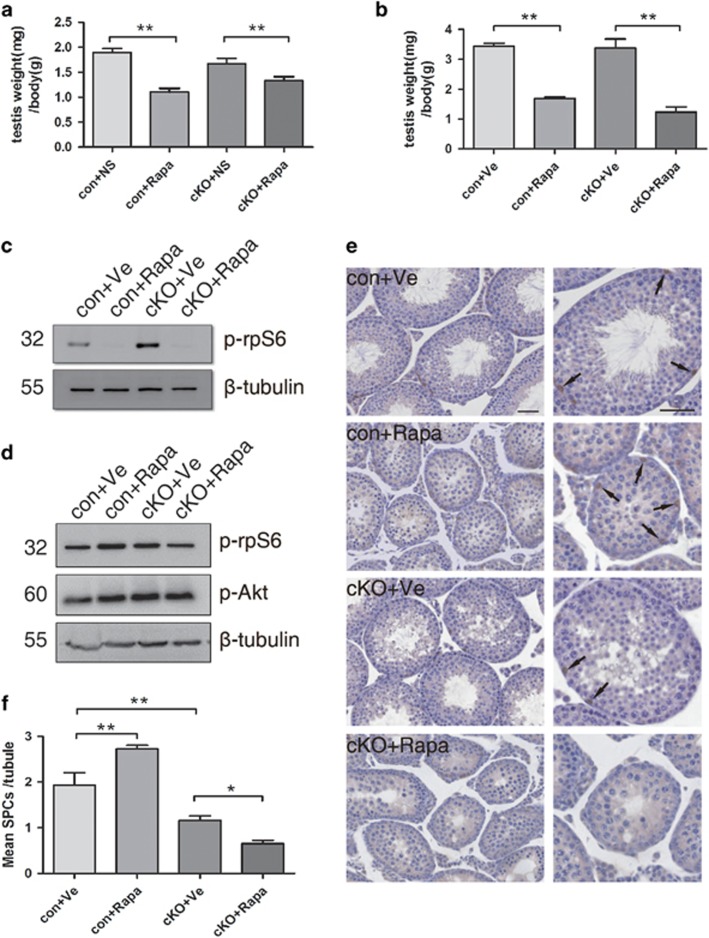
Rapamycin treatment failed to rescue the depletion of SPCs in the tubules of *Lkb1* cKO mice. Rapamycin was given to P10 control and cKO mice (*n*=5 in each group) for 1 week, and testes were evaluated at 5 weeks of age. con+Ve, control mice injected with vehicle control saline; con+Rapa, control mice injected with rapamycin; cKO+Ve, cKO mice injected with vehicle control; cKO+Rapa, cKO mice injected with rapamycin. (**a**) Testis weight per body weight just after injection. (**b**) Testis weight per body weight at 5 weeks of age. (**c**) Western blot analysis of p-rpS6 level just after injection. (**d**) Western blot analysis of p-rpS6 and p-Akt level at 5 weeks. **(e**) Seminiferous tubules immunostained for Plzf. Scale bar=50 *μ*m. Images on the right column are higher magnifications of the relative tubules in the left. Arrows, representative Plzf-positive SPCs in the tubules. (**f**) Average number of Plzf-positive cells per tubule in each of the four groups. Data presented as mean±S.E.M. of at least three replicates. *P<0.05, **P<0.01

Next, we assess whether rapamycin (the mTORC1 inhibitor) could reverse the loss of SSCs in cKO mice. We use testes just after injection to test Rapamycin effect and collect testes at 5 weeks of age for analysis. As expected, rapamycin treatment caused a significant decrease of testis per body weight in both control and cKO mice compared to that in vehicle-control mice (Figures 8a and b). The level of p-rpS6 was dramatically reduced after injection and recovered to normal at 5 weeks of age (Figures 8c and d). In control mice of the rapamycin-treated group, the seminiferous tubules displayed a reduced diameter and a blockade of germ cell development at stages earlier than spermiogenesis (Figure 8e, control+Rapa). Moreover, the number of Plzf-positive germ cells increased ~1.4-fold when compared with control vehicle-treated control testes (Figure 8f). However, rapamycin treatment in cKO mice did not have a rescue effect on SPCs accumulation in cKO mice (Figure 8e, cKO+Rapa). In fact, the number of Plzf-positive cells was slightly below that of vehicle-treated cKO testes (Figure 8f). We infer from these results that although the deletion of *Lkb1* in germ cells will yield an increase in the number of differentiating spermatogonia via upregulation of mTORC1 activity, the regulation of Lkb1 on SSC self-renewal or maintenance is mTORC1-independent.

Since mitochondria dysfunction is associated with the failure of stem cell maintenance in *Lkb1*-deficient hematopoietic stem cells (HSCs),^41^ we next test the mitochondrial membrane potential (ΔΨm), a sensitive and reliable indicator of mitochondrial activity, in *Lkb1*-deficient SPCs by JC-1 staining.^42^ After a ratiometric analysis of JC-1 staining, a significant reduction in ΔΨm was observed in SPCs of *Lkb1* cKO mice (Supplementary Figures S2a,b). Thus, instead of mTOR signaling pathway, the regulation of LKB1 on mitochondrial function may be essential in SSC maintenance and survival.

## Discussion

Herein we showed that *Lkb1*_*L*_ and *Lkb1*_*S*_ functioned coordinately in regulating spermiogenesis, while *Lkb1*_*L*_ also served as the key regulator for SSC maintenance. Previous studies showed that *Lkb1*_*S*_ KO male mice were infertile with defects in the release of mature spermatids.^8^ Moreover, *Lkb1*_*S*_-deficient spermatozoa were immotile and presented head and tail morphological abnormalities.^7^ In our study, deletion of both *Lkb1* isoforms from germ cells also resulted in sterility but more severe defects in spermiogenesis. Firstly, in addition to the coiled tails or round heads observed in spermatozoa from *Lkb1s* KO mice, most abnormal spermatozoa found within the epididymis of *Lkb1* cKO mice were multinuclear and multiflagellar, something seldom seen in *Lkb1*_*S*_ KO mice. Secondly, the most striking difference between *Lkb1*_*S*_ KO and *Lkb1* cKO testes was that the latter lacked elongated and condensing spermatids in their seminiferous tubules at stages VIII–XII. Further experiments showed that the severe defects on acrosome formation happened as early as the beginning of the Golgi phase, which likely precluded the establishment of nuclear polarity and ultimately resulted in the failure on nucleus elongation and flagellar formation.^13, 43^ There is growing evidence that LKB1 and AMPK play pivotal roles in the establishment of cell polarity in epithelial cells,^44, 45, 46, 47^ intestinal paneth and goblet cells,^4^ and pancreatic *β*-cells.^48, 49^ Recent studies show that a cytoplasmic linker protein of 170 kDa, CLIP-170, functions downstream of AMPK to affect cell polarity by shifting its binding sites to the more distal ends of microtubules.^50^ In mice, CLIP-170 has been identified to associate with the sperm manchette, and CLIP-170 knockout mice were subfertile and produced sperm with abnormal heads.^51^ Therefore, it is feasible that the same regulatory mechanism functions for LKB1-mediated nuclear polarization during spermatid differentiation. Despite tissue-specific differences, both *Lkb1*_*L*_ and *Lkb1*_*S*_can activate AMPK and AMPK-related kinases *in vitro*.^7^ However, given the different phenotypes in spermiogenic defects observed, further studies are required to understand their downstream effectors in the process of spermiogenesis.

Spermatogenesis – the differentiation of male germ cells – is a specialized developmental process, which is precisely regulated at the transcriptional, posttranscriptional, and translational levels.^52, 53^ Following meiosis, the beginning of spermiogenesis is characterized by a massive wave of transcription in round spermatids and subsequent expression of genes are required for morphological and biochemical reprogramming.^54^ To date, only a limited number of genes were identified as key regulators of spermiogenesis, including *Crem*, *Rnf17*, *Tpap*, *Ddx25*, *Trf2*, *Rfx2*, *Piwil* and *Boule*.^15, 16, 17, 18, 19, 20, 21, 22, 23, 24^ Accordingly, our results suggest a selective downregulation of these key regulators during spermiogenesis in cKO testis. However, the decreased gene expression levels stemming from *Lkb1* cKO testis provided some but not sufficient support for the completion of spermiogenesis.

The detrimental effects of aberrant activation of mTORC1 in stem cell maintenance have been extensively reported.^35, 39, 55^ Moreover, the important roles of mTORC1 in SSC differentiation have emerged in recent years.^56, 57, 58, 59^ As the major upstream kinase to phosphorylate AMPK, LKB1 is best known to inhibit mTORC1 activity through activation of AMPK.^2^ We inferred aberrant activation of mTORC1 results in accelerating SSC differentiation. However, our following experiments using *in vivo* rapamycin treatment failed to rescue the defects on SSC maintenance. Furthermore, to some extent, this treatment slightly accelerated germ cell depletion in *Lkb1* cKO mice. Therefore, based upon these results, the effects on SSC differentiation stemming from *Lkb1* deletion cannot be solely due to a downstream effect on the mTOR signaling pathway, and other mechanisms must be in place in the regulation of LKB1 on SSC maintenance and survival.

As an evolutionarily conserved regulator of cellular energy metabolism, the involvement of LKB1 in stem cell maintenance has been only reported in HSCs.^41^ Inactivation of *Lkb1* in mice caused progressive depletion of HSCs and eventual pancytopenia. Although the deletion of *Lkb1* was associated with predictable loss of p-AMPK*α* and an increase in p-rpS6, it was shown that *Lkb1* regulation of HSC maintenance occurred in an AMPK- and mTORC1-independent manner.^41, 60, 61^ Concurrently, the *Lkb1*-deficient HSCs exhibited mitochondrial defects, aneuploidy and ATP depletion.^41, 60, 61^ In our study, survival and maintenance of SSCs was also in an mTORC1-independent manner. And, a significant decrease of mitochondrial activity was observed in *Lkb1*-deficient SPCs. A stem cell niche was essential for maintenance of stem cell populations.^62^ Although this niche may be essentially different between HSCs and SSCs, similar phenotypes suggest that a common mechanism exists in the regulation of *Lkb1* in adult stem cell homeostasis. It would be very beneficial to define the common and specific effectors when comparing the role of LKB1 in these two stem cell types. Since *Lkb1*_*L*_ is the major isoform expressed in SSCs, our results revealed its indispensable role on maintenance of the stem potential in SSCs.

In summary, by using *Lkb1* cKO mice, we demonstrated the differential regulation mediated by *Lkb1* isoforms in spermatogenesis. Although *Lkb1*_*S*_ is the dominant isoform expressed in testis and plays crucial roles in spermiogenesis, the *Lkb1*_*L*_ was also required, with both isoforms functioning independently or coordinately at different stages of spermatogenesis. Our data suggest an indispensible role of *Lkb1*_*L*_ in SSC maintenance and the cooperative regulations of both *Lkb1* isoforms in spermatid differentiation.

## Materials and methods

### Mouse genetics, husbandry and treatment

Mice carrying floxed Lkb1 alleles (FVB; 129S6-Stk11tm1Rdp/Nci) were obtained from NCI Mouse repository. Vasa-Cre mice were purchased from Model Animal Research Center of Nanjing University (Nanjing, Jiangsu, China).^63^ The floxed Lkb1 mouse strain was backcrossed onto a C57BL/6 background for at least nine times. The two strains were kept on the same genetic background (C57BL/6) and were bred by standard husbandry techniques to obtain conditional knockout mice with deletion *Lkb1* gene in germ cells only (*Lkb1* cKO mice). The primers to detect wt and flox Lkb1 alleles are R1: 5′-CTGTGCTGCCTAATCTGTCG-3′, F2: 5′-TTCACCATCCCTTGTGACTG-3′ and F4: 5′-ATCGGAATGTGATCCAGCTT-3′.^60^ The mice were housed in the animal facility at Nanjing Medical University and all animal protocols were approved by the Committee on the Ethics of Animal Experiments of Nanjing Medical University. Testes at different developmental stages (P8, P24, 5 W, 6 W, 13 W, 20 W and 40 W) were collected to evaluate morphology and other analyses.

To test the effect of rapamycin on SSC maintenance and differentiation, mice at P7 from control and *Lkb1* cKO mice were treated daily with rapamycin (4 mg/kg, LC Laboratories, Woburn, MA, USA) or vehicle for 7 days as described^39^ and then raised until 5 weeks of age for testes collection.

### Germ cell isolation and SSC culture

Germ cell isolation and SSC culture were established according to previous study.^28^ Briefly, germ cells were isolated from P8 of control and cKO mice and Thy-1-positive cells were enriched by using magnetic activated cell sorting (Miltenyi Biotech, Bergisch Gladbach, Germany). Cells were plated at a density of 1.5 to 2 × 10^5^ per well on 12-well plates with mitotically inactivated STO feeder layers (ATCC, Manassas, VA, USA). Medium was replaced every 2–3 days and were passaged at a ratio of 1 : 2 every 5–6 days. Cell numbers were counted at each passage in order to plot the cell growth curve. Cultures were maintained at 37 °C in an incubator with humidified 5% CO_2_ and 95% air atmosphere.

### Sta-put velocity sedimentation

Spermatogenic cell fractionation was performed by sedimentation of cells prepared from adult mouse testes through a BSA gradient as previously described with a modification.^64^ At least 30 testes were pooled from male mice at 5 weeks of age and incubated with 1 mg/ml collagenase IV in DMEM (Life Technology, Waltham, MA, USA) for 15–20 min at 37 °C in water bath and gently shook into seminiferous tubules. The cords were then washed twice in DMEM by sedimentation at unit gravity and decanting the supernatant. Further digestion were then performed with 1 mg/ml DNase I in 0.25% trypsin for 5–15 min at 37 °C. Pellet cells at 500 g for 5 min and re-suspend pellet with wash buffer (10%FBS, 0.5% BSA, 200 *μ*g/ul DNase I). Monodisperse cell suspension was made after filtering the cells with 40 *μ*m cell strainer. The dispersed cells were layered over a linear gradient (2–4%) of BSA in DMEM, then allow to sediment at unit gravity for a total period of 2.5–3 h. The cells were collected, identified and pooled into quasi-homogeneous populations. The isolated cell types were characterized on the expression of marker genes by RT-PCR.

### Sperm counts and sperm staining

Adult wild-type and *Lkb1* cKO mice (13 weeks of age) were killed and one caudal epididymidis was dissected from each mouse to collect spermatoza. The extruded spermatoza were incubated in 37 °C PBS for 15 min and the suspension was diluted 1 : 100 for sperm counting using a hemocytometer. After cell counting, 10 *μ*l of the diluted suspension was collected to draw a sperm smear. Then the smears were stained with eosin for morphological examination.

### Immunohistochemistry and mmunoflurescence

Mouse testes were fixed in Hartman’s Fixative (Sigma, MO, USA; H0292) overnight before embedding in paraffin. After deparaffinization and rehydration, tissue sections were then incubated for 15 min in 3% (v/v) hydrogen peroxide in methanol to block endogenous peroxidase activity and antigen retrieval was pretreated by boiling the tissue sections in 0.01 M citrate buffer for 15 min. Immunohistochemical analysis were performed using a SP-link Detection Kit (Zhong Shan Jin Qiao, Beijing, China; SP9001 for anti-rabbit, SP9002 for anti-mouse) with primary antibodies overnight at 4 °C. Sections were counterstained with hematoxylin following detection. Primary antibodies used were as follows: p-rpS6 (CST, Cell Signaling Technology, Beverly, MA, USA; catalog no. 4858; 1 : 800) (CST,), PCNA (CST; catalog no. 13110; 1 : 16000), Lin28a (Abcam, Cambridge, MA, USA; catalog no. 46020; 1 : 2000) and Plzf (R&D Systems, Minneapolis, MN, USA; catalog no. AF2944; 1 : 500). Immunofluorescence was performed with antibodies for VASA (Abcam; catalog no. 13840; 1 : 400), GATA4 (Santa Cruz, CA, USA; catalog no. 25310; 1 : 100), *γ*H2AX (Abcam; catalog no. 26350; 1 : 400) and p-rpS6 (CST). After incubation overnight at 4 °C, the primary antibodies were washed out and sections were incubated with relative secondary antibodies at RT for 1 h. The secondary antibodies include Alexa Fluor 488 donkey anti-rabbit (Invitrogen, Grand Island, NY, USA; catalog no. A21206; 1 : 500), Alexa Fluor 594 donkey anti-mouse (Invitrogen; catalog no. A21203; 1 : 500), Alexa Fluor 488 goat anti-mouse secondary antibodies (Invitrogen; catalog no. A21202; 1 : 500). Then the nuclei were stained with 0.01 mg/ml Hoechst 33342 (Invitrogen; catalog no. H1339) for 20 min and sections were viewed under a laser scanning confocal microscope (LSM 510 META, Zeiss, Germany).

### Acrosome staining

Testes at 6 weeks of age were collected in both control and cKO mice. Tissues were fixed in Hartman’s Fixative overnight, then incubated in 5 and 30% sucrose, embedded in optimum cutting temperature compound (OCT, Tissue-Tek, Torrance, CA, USA) and cut into 8 *μ*m sections using a microtome-cryostat (Thermo Cryotome FSE). Frozen sections were washed in PBS three times and permeabilized in 0.1% Triton X-100 for 10 min. After washing in PBS, sections were incubated with Fluorescein isothiocyanate-conjugated peanut agglutinin (FITC-PNA, Sigma; catalog no. L7381; 1 : 500) at a final concentration of 10 *μ*g/ml for 1 h at RT. The nuclei were counterstained with Hoechst 33342 and viewed under a laser scanning confocal microscope (LSM 510 META, Zeiss, Germany).

### TUNEL assay

Testes were collected from three different control and mutant mice and fixed overnight at RT in Hartman's Fixative (Sigma). TUNEL assays were performed on 5 *μ*m sections with the *In Situ* Cell Death Detection Kit (Roche Applied Science, Indianapolis, IL, USA; catalog no. 12156792910) according to the manufacturer’s instructions.

### Western blots

Testes from control and *Lkb1* cKO mice were collected and proteins were extracted by RIPA lysis buffer (Beyotime Institute of Biotechnology, Shanghai, China; catalog no. P0013B) with protease inhibitor cocktails (Amresco, Solon, OH, USA; catalog no. M221). After electrophoresis and electronic transfer, the membranes were blocked in 5% skimmed milk-TBST (TBS containing 0.1% Tween 20) for 30 min and then incubated overnight at 4 °C with specific antibodies. The following antibodies are used: LKB1 (CST; catalog no. 3047; 1 : 1000), p-S6K1 (phosphorylated at Thr389; CST; catalog no. 9234; 1 : 1000), rpS6 (CST; catalog no. 2217; 1 : 1000), p-rpS6 (CST), *β*-tubulin (CST; catalog no. 2128; 1 : 2000), Lin28a (Abcam; catalog no. 46026; 1 : 2000), Plzf (R&D; catalog no. AF2944; 1 : 1000), Akt (CST; catalog no. 2920; 1 : 2000) and p-Akt (CST; catalog no. 4060; 1 : 2000). Horseradish peroxidase-conjugated goat anti-rabbit (Zhong Shan Jin Qiao; catalog no. ZB2301; 1 : 2000), goat anti-mouse (Zhong Shan Jin Qiao; catalog no. ZB2305; 1 : 2000), rabbit anti-goat IgGs (Zhong Shan Jin Qiao; catalog no. ZB2306; 1 : 2000) were then used to detect proteins through enhanced chemiluminescence (Amersham, Pittsburgh, PA, USA).

### Transmission electron microscopy (TEM)

Adult caudal epididymidis (13 weeks) or testes at 6 weeks of age were fixed with 2.5% glutaraldehyde in 0.2 M cacodylate buffer overnight and cut into small pieces (1 mm^3^) after washing in 0.2 M cacodylate buffer. Small blocks were dehydrated through a graded ethanol series and embedded in resin. Ultrathin sections were cut on an ultramicrotome and observed with a transmission electron microscope (Tecnai G2 Spirit Bio TWIN).

### Quantitative RT-PCR analysis

RNA was extracted from testes or germ cells using TRIzol reagent (Invitrogen) according to the manufacturer’s instructions. Reverse transcription was performed on 500 ngRNA using HiScript II qRT super mix (Vazyme, Nanjing, China). Quantitative real-time PCR was performed using Eva green master mix (Applied Biological Materials Inc, Vancouver, Canada) on an ABI StepOne™ Real-Time PCR System (Applied Biosystems, Foster City, CA, USA). The relative expression of each gene was calculated by a 2-ΔCT method with the expression of *β*-actin as internal control. All the primers for RT-PCR were seen in Supplementary Table S1.

### Measurement of mitochondrial membrane potential (ΔΨm)

To measure the mitochondrial membrane potential, SPCs were incubated for 20 min at 37 °C with 5% CO_2_ in SSC culture medium supplemented with 2 *μ*M JC-1 probe (Beyotime Institute of Biotechnology, Beijing, China), in accordance with the manufacturer’s protocol. After washing twice, SPCs were analyzed immediately by confocal microscopy (LSM 700, Zeiss, Germany). JC-1 is a cationic dye that produces two fluorescence emission peaks, indicated by a fluorescence emission shift from green (JC-1 monomers) to red (JC-1 aggregates). Consequently, mitochondrial depolarization was indicated by a decrease in the red/green fluorescence intensity ratio.

### Statistical analysis

All data are presented as the mean±S.E.M. and the statistical significance of the difference between control and cKO mice were examined using Student’s *t*-test with a paired two-tailed distribution. The data were considered significance when *P*<0.05 (*) or 0.01(**). Values were calculated by using Prism 5.0 for Macintosh (GraphPad Software, Inc., La Jolla, CA, USA).

## Publisher’s Note

Springer Nature remains neutral with regard to jurisdictional claims in published maps and institutional affiliations.
