# Supplementary material for: Differential regulation of spermatogenic process by Lkb1 isoforms in mouse testis
Source: Cell Death Dis. 2017 Oct 12;8(10):e3121–. doi: 10.1038/cddis.2017.527 (PMC5682689; doi:10.1038/cddis.2017.527)
Supplement: Supplementary Information [file cddis2017527x1.doc]

Supplementary Figures, Figure Legends


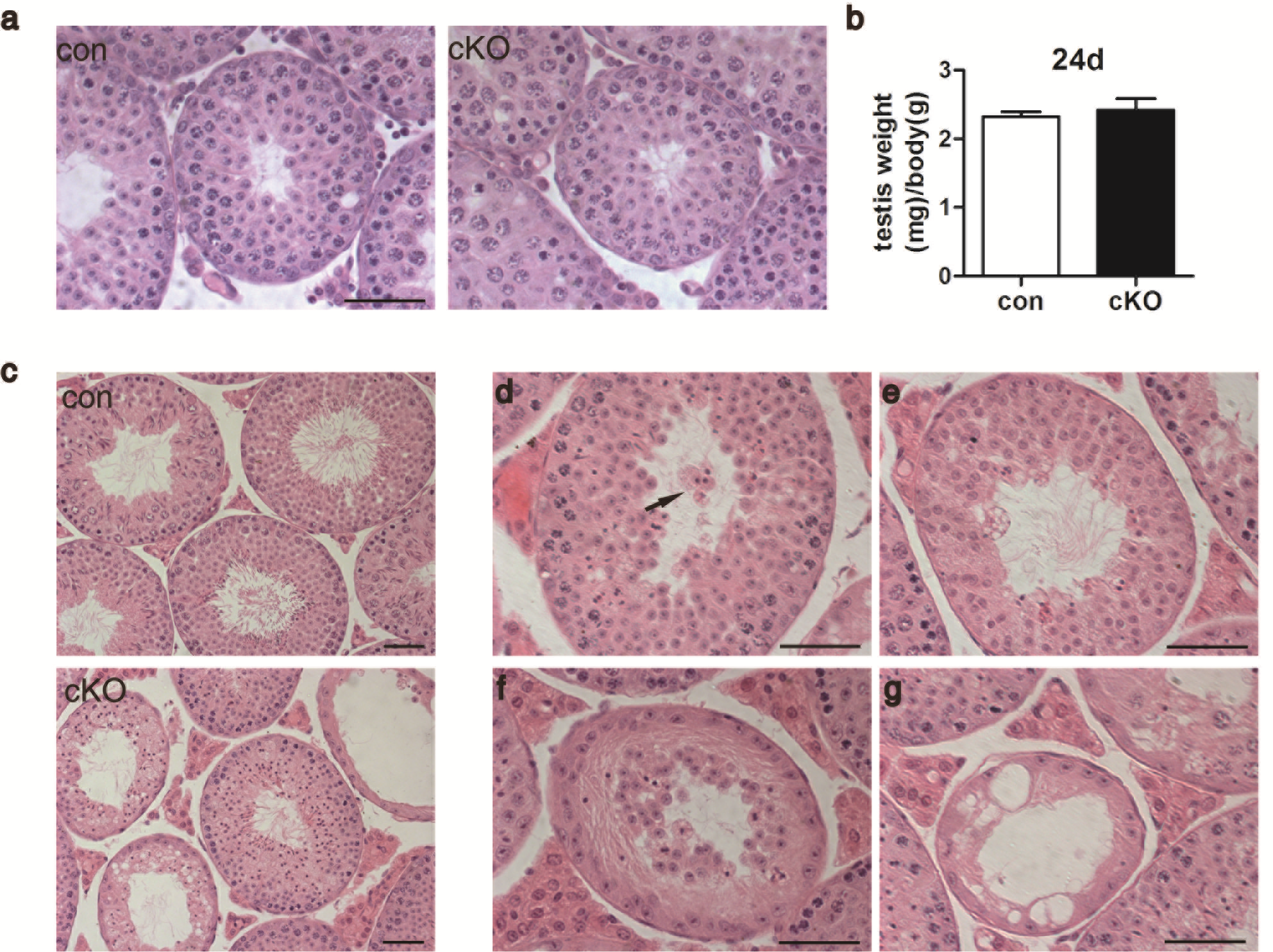


**Figure S1.** Histological analysis of testes at P24 and 13 weeks of age in control and cKO mice (n=3). (a, b) There was no obvious difference on testis morphology (a) or relative weight (b) between control and cKO mice at P24. (c) Comparison of testicular tubules in control and cKO mice at 13 weeks age. Tubules undergoing [varying](https://cn.bing.com/dict/clientsearch?mkt=zh-CN&setLang=zh&form=BDVEHC&ClientVer=BDDTV3.5.1.4320&q=不同程度) [degrees](https://cn.bing.com/dict/clientsearch?mkt=zh-CN&setLang=zh&form=BDVEHC&ClientVer=BDDTV3.5.1.4320&q=不同程度) of germ cell loss were observed in *Lkb1* cKO testis. (d-g) Higher magnification of representative tubules shown in *Lkb1* cKO testis (c). (d) A tubule with almost a normal number of germ cells; (e) A tubule with no spermatocytes; (f) A tubule with several spermatids left; (g) An empty tubule with only Sertoli cells. Scale bars=50 μm. Arrow, sloughed cells in the centre of the tubule.


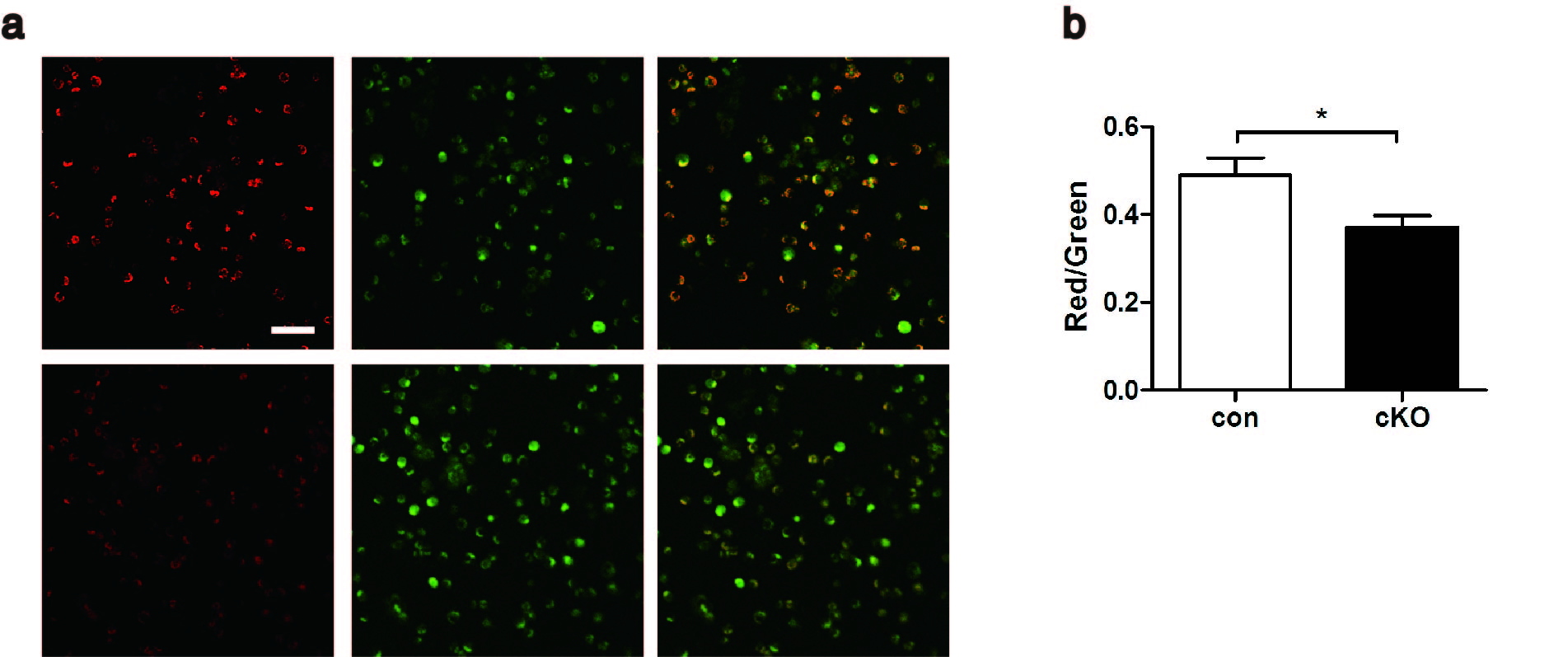


**Figure S2.** The reduction of mitochondrial membrane potential (ΔΨm) in *Lkb1* deficient SPCs. (a) Representative JC-1 staining of SPCs isolated freshly from con and cKO mice. Red, JC-1 aggregates; green, JC-1 monomers. (b) ΔΨm was reflected by the ratio of red/green fluorescence intensity. Data were shown as mean±S.E.M of at least three replicates. *P < 0.05.

Table S1 Primers for Real-time PCR

| **Mouse gene name** | **Forward** | **Reverse** |
| --- | --- | --- |
| *Lkb1_L_* | CCTGCAAGCAGCAGTGAC | CCAACGTCCCGAAGTGAG |
| *Lkb1_S_* | CATTATCTACACCCAGGACTTCACA | CGCATGCATCCTCGCTAA |
| *Tnp1* | ACCAGCCGCAAGCTAAAGAC | TTTCCTACTTTTCAGGACGCTC |
| *Sycp2* | GACACTGAAACCGAATGTGGA | GACACTGAAACCGAATGTGGA |
| *Plzf(Zbtb16)* | CCCTATGAGTGTAATGGCTGTG | TTCTCAGGTGCTTGATCATGG |
| *Crem* | ATGTCTTGAAAATCGTGTGGCT | TGGCAATAAAGGTCTTTGAGGG |
| *Rnf17* | CACCTAGTGGAGAGTAAGAGTGG | TGCTGACAGTTGTATGAACCTC |
| *Tpap* | AGCATCCACTCTTGTGCGAAA | GGTCCCATACAGGCAAGTTAAG |
| *Ddx25* | ATGGCGTCGTTACTTTGGGG | AGAGCCGTCTATGTTTGGGAC |
| *Boule* | TTTGGTGCCAGACGTTTAGC | GCCACAGCCTTTACATTGGG |
| *Rfx2* | AGACCCTCAGCTTTACGCC | GTGCCACCTGGAGTCTCAAA |
| *Trf2* | TTTGGTGCCAGACGTTTAGC | GCCACAGCCTTTACATTGGG |
| *Piwil* | AGACCTCATTGGAAGGTGTCA | TGTTCCCCATTCCGAGTCTGA |
| *Etv5* | CACCATGTATCGAGAGGGGC | GAGCAACCTCTTCCGGTTCT |
| *Bcl6b* | CGGAGCACGTTTTAACCGAC | CAGGGGTCACAGTGGTATGG |
| *Lin28a* | AGA TGC TCA AAG AAG TAA ATG | TCC TCT TCT CAA AGC GAA CCT |
| *Id4* | CGTTATCGACTACATCCTGGAC | TCTTAATTTCTGCTCTGGCCC |
| *Nanos3* | AGA TGC TCA AAG AAG TAA ATG | TCC TCT TCT CAA AGC GAA CCT |
| *Oct4(Pou5f1)* | TAGGTGAGCCGTCTTTCCAC | GCTTAGCCAGGTTCGAGGAT |
| *Stra8* | TTAAACCAGGAACCAGAGC | TTAAACCAGGAACCAGAGC |
| *c-kit* | TGTGGCTAAAGATGAACCCTC | ACACTCCAGAATCGTCAACTC |
| *Taf4b* | AGA TGC TCA AAG AAG TAA ATG | TCC TCT TCT CAA AGC GAA CCT |
| *Gfrα1* | CTC GGA ATC CAG CCT ACG TC | CAC TTG TCC TCT CGT GTG CT |
| *Ret* | GCATGTCAGACCCGAACTGG | CGCTGAGGGTGAAACCATCC |
| *Sohlh1* | CAT TAC GGG ATG CAG CAA GAC | GAC AGA TGC CAT GTC CTC CC |
| *Sohlh2* | CCG TGC TTT TGG CTG CTA AC | TCA CTG AAA CTA ATG TCA GCT CC |
| *Actin* | CCGTAAAGACCTCTATGCC | CTCAGTAACAGTCCGCCTA |
